# Developing and standardizing a tool to assess the health education needs of diabetic patients at Jazan Armed Forces Hospital

**DOI:** 10.1186/s42506-025-00183-1

**Published:** 2025-02-18

**Authors:** Hassan A. Abdelwahid, Hesham M. Dahlan, Gassem M. Mojemamy, Turki J. Al-Harbi, Nouf Y. Indarkiri, Ayla M. Tourkmani

**Affiliations:** 1 Family Medicine Department, Faculty of Medicine, Suez Canal University, Ismailia, Egypt; 2 Family Medicine Department, Jazan Armed Forces Hospital (JAFHS), Jazan, Saudi Arabia; 3 Family Medicine Department, Prince Sultan Military Medical City (PSMMC), Riyadh, Saudi Arabia; 4 PSMMC, Riyadh, Saudi Arabia

**Keywords:** Diabetes, Health, Education, Likert, Scale, Questionnaire

## Abstract

**Background:**

Determining the health educational needs of people living with diabetes is essential in developing patient-centered, structured health education programs that aim to improve the outcome of diabetes care.

**Objectives:**

To develop a tool for the identification of the health education needs of individuals living with diabetes in the Jazan Armed Forces Hospital (JAFH) and to standardize the questionnaire through the assessment of its reliability and validity.

**Methods:**

A cross-sectional design was used in the present work, which included 303 participants living with diabetes. The researchers and an expert panel in family medicine and endocrinology created a comprehensive and mutually exhaustive questionnaire covering every potential area of health education needs. It included a 15-item section with questions on a 5-point Likert scale for determining the participants’ needs for health education. Cronbach’s alpha was used to determine the Likert scale’s reliability. Exploratory factor analysis was used to determine the Likert scale’s construct validity.

**Results:**

The total number of males was 123 (40.6%) and that of females was 180 (59.4%). Their mean ages were 55.9 ± 12.9, ranging from 18 to 94 years. The reliability of the 15-item Likert scale was 83%, and it increased to 90% when the redundant items (*n* = 5) were eliminated. The test had an 86% test–retest reliability when repeated. Also, the final 10-item Likert scale has significant face, content, and construct validity. Two components with eigenvalues over 1 (generic knowledge about diabetes, and diabetes and travel) could be extracted out of the 10-item Likert scale.

**Conclusion:**

The final 10-item Likert scale offers a good degree of validity and reliability for determining the health education needs of individuals living with diabetes. The two Likert scale components (general information on diabetes, and diabetes and travel) and their contributing items were identified from the questionnaire, which is standardized and helpful in both practice and research, in order to ascertain patients' needs and develop structured health education programs. The component “General information about diabetes” exhibited significant associations with the following items: diabetes risk factors and prevention; common oral agents for treating hypoglycemia; HbA1c (glycosylated hemoglobin) and normal blood glucose levels; and acute problems related to diabetes, such as hypoglycemia and diabetic ketoacidosis. On the other hand, diabetes and fasting; chronic complications of diabetes; and the significance of the yearly eye screening were the Likert scale items that contributed more to Component 2 (diabetes and travel).

## Introduction

Diabetes is a dangerous and intricate metabolic illness that has a significant impact on the lives of those who have it as well as their families. Diabetes in kids and teens can make it more difficult for families to function and impede their normal social and psychological growth [1]. Its incidence is becoming epidemic-like on a worldwide basis. According to the International Diabetes Federation, about 537 million adults (20–79 years) are living with diabetes worldwide and it is projected to increase to 643 million by 2030 and 783 million by 2045 [2].

Diabetes is a serious disease, as are its associated consequences. Nonetheless, there is compelling evidence that prediabetes, type 2 diabetes, and diabetic complications can be avoided, postponed, or have their progression reduced through glycemic management, lifestyle changes, appropriate medication administration, and control of risk factors like hyperlipidemia and hypertension. Additionally, there is evidence that type 2 diabetes can be remitted with the use of preventative treatments [3].

Preventive measures can assist patients in managing their diabetes. Nevertheless, a large number of diabetic patients struggle with weight loss, smoking cessation, and inadequate blood pressure, cholesterol, or blood sugar control. In the USA, about one-third of adult diabetics do not have the appropriate HbA1c readings. Even fewer have good glycemic control, ideal blood pressure, and serum cholesterol levels. Therapeutic targets are not being fulfilled in diabetes care, despite numerous advancements [4].

An essential component of managing diabetes is diabetes self-management education and training [4]. Patients with diabetes should understand the causes, symptoms, risks, and treatments associated with their condition. It is crucial to raise community awareness of the unique requirements of those who live with diabetes [5]. Thankfully, patient education and focused interventions have the power to change things. Diabetes self-management education and support (DSMES) is recommended by professional associations such as the American Diabetes Association (ADA). Evidence-based diabetes self-management education and support is a procedure that takes into account the requirements, and life experiences of each patient with diabetes [6]. To give diabetic patients the information, skills, and support they need to self-manage their condition, diabetes self-management education and support takes a broad approach rather than a set of detailed recommendations. The following are the main tenets of DSMES: (1) diabetes self-management education and assistance is a continuous process. (2) All diabetic patients should be made aware of the advantages and importance of both initial and ongoing DSMES by their providers [6].

In addition to nutrition, exercise, medications, and insulin therapy, diabetes education is a crucial part of the treatment plan [7]. Patients benefit from education in many ways, including increased knowledge and skills, altered behaviors, improved quality of life, increased motivation to follow treatment instructions, increased awareness of cardiovascular risk factors, increased psychological flexibility, and preparedness to practice self-care [8]. The following are the key topics for diabetes patient education: (1) evaluation of the patient’s familiarity with diabetes; (2) introduction to the pathological mechanisms underlying the disease; (3) nutritional issues; (4) exercise; (5) smoking cessation; (6) blood glucose monitoring; (7) medication use that is both safe and efficient; (8) acute complications; and (9) chronic complications [8, 9].

A thorough needs assessment is the initial stage in the design and production of educational materials. The information gathered from these needs assessments is the foundation of every intervention program and is used as a basis for curriculum construction to raise the skills and knowledge of patients [10]. Developed as a knowledge acquisition tool, the Delphi method offers a structured mechanism for anticipating and supporting decision-making during survey rounds of information gathering, leading to group consensus. Understanding patients’ needs from their perspectives and in their own words allows for a more complete and unbiased view of each person's individual experiences [11].

No published study has created a standardized instrument to evaluate patient needs in relation to type 2 diabetes health education. Nonetheless, in a three-round qualitative study carried out in Iran in 2017–2018 which made use of a modified Delphi technique, the authors came to the conclusion that there were four major themes pertaining to the healthcare needs of patients with diabetes: the need for information and education, the need for religious and cultural beliefs to be reinforced or changed, the need for health self-management, and finally the need for supportive measures [12, 13].

According to the Declaration of Alma-Ata, 1978 [14], one of the key objectives of primary health care is to educate patients and families about common health issues and how to prevent and control them through the use of scientifically sound methods and technology. Family physicians are best suited to offer structured diabetes health education programs. Determining the health and educational needs of people with diabetes is essential for developing patient-centered programs that will enhance the treatment of diabetes. This study aims to standardize a method for assessing health education needs based on patients’ experiences based on the perspective of both patients and healthcare professionals.

## Materials and methods

### Study design and setting

A cross-sectional design was used in the present work that was conducted at Jazan Armed Forces Hospital (JAFH). Jazan is the capital city of the Jazan region lies in the southwest corner of Saudi Arabia on the Red Sea coast, just north of Yemen, and has a large agricultural community. The JAFH is a 100-bed secondary hospital that provides health care for military personnel and their families (approximately 100,000).

### Sample size justification and sampling procedure

The sample size was calculated according to the following cross-sectional formula: [15]

$$n\geq\;P\left(1-P\right)\;\left(Z/D\right)^2$$

Where.

*n* = sample size; *Z*_α/2_ = 1.96 (1.96 for 95% confidence level); *P* = the prevalence (from a previous study = 16.4% [16]; and *D* = confidence interval (CI), expressed as decimal (0.05). The nonresponse was considered to be 10%, so the sample size should be ≥ 232.

Every third patient, based on their chronological attendance at the reception desk in various JAFH outpatient clinics, was chosen for the study using a systematic random sample technique.

### Inclusion criteria

1. People living with diabetes.
2. Eligibility for medical care at JAFH.
3. Consent to participate in the survey.

### Exclusion criteria

Patients presenting with serious diabetic complications or having severely uncontrolled diabetes.

### Tools of the study

The researchers and a team of 20 experts in endocrinology and family medicine created the tools.

#### Sociodemographic questionnaire

The first questionnaire was designed to collect sociodemographic data like participants’ age, sex, type and duration of diabetes, the source of the health education they thought was best, etc.

#### Likert scale questionnaire for educational needs assessment

This Likert scale questionnaire contained 15 statements organized in a 5-point Likert scale format to ascertain the participants' health education needs. The Likert scale is an ordered scale that is best suited for assessing attitudes, views, and perceptions in research [17]. The questionnaire was developed methodically using a modified Delphi panel procedure: [18, 19].

##### Focus group discussion

The researchers (group discussion participants, *n* = 5) who are experts in family medicine provided the suggested statements of the Likert scale questionnaire, which included 17 items, based on an overview of pertinent literature. The researchers got together in person.

##### Modified Delphi method (two rounds)

In the first round, a panel of 20 experts in the domains of endocrinology and family medicine, who were not members of the research team, were asked to rate their opinions on the 17-item questionnaire, that was suggested by the researchers. To confirm anonymous comments, the panelists' replies were acquired through a website specifically designed for this purpose or emails for those who did not access the website. In the first round, the panelists’ agreement to identify the statement as a need for health education for people living with diabetes was indicated by their response if they chose to agree or strongly agree. Responses that indicated a strong disagreement, disagreement, or neutrality were taken as evidence that the health education statement in question was not necessary. A questionnaire consisting of 15 items was created by eliminating statements that had a consensus rate of less than 70%. These statements included “lipid profile normal range” and “atherosclerotic cardiovascular disease risk in diabetes.” In the 2nd round, the panelists, *n* = 20, were requested to assess the 15-item questionnaire that resulted from round 1. The second round of questionnaires gave participants the opportunity to see how the rest of the group prioritized areas and to see if they wanted to change their opinion on the basis of the group consensus. All statements had a consensus rate of ≥ 70% and were retained in the final Likert scale questionnaire that included 15 statements.

The statements of the Likert scale questionnaire were: risk factors and prevention of diabetes; prediabetes (causes, symptoms, and treatment); general information about diabetes (pancreas and insulin, causes of diabetes, diagnosis, symptoms, and types); HbA1c and normal blood glucose levels; treatment of hypoglycemia with common oral agent; medical treatment with insulin (types, storage, sites of injection, and insulin pump); diabetes and complementary therapies; acute complications of diabetes (hypoglycemia, diabetic ketoacidosis, etc.); chronic complications of diabetes, causes, symptoms, and treatment; the importance of annual eye screening to prevent diabetic eye complications; diabetes and travel; diabetes and fasting; diabetes and pilgrimage; diabetes and work and driving; and diabetes and school.

All potential areas of the need for health education were covered by the Likert scale statements. From strongly disagreeing (not significant at all) to disagreeing (significant) to neutral (don’t know) to agreeing (important) to strongly agreeing (extremely important), the participants’ responses varied. The 5-point Likert scale was translated into scores ranging from 1 for strongly disagree to 5 for strongly agree because every statement on the Likert scale was a positive one. The entire Likert scale questionnaire had scores ranging from 15 to 75, whereas the statements’ scores fell between 1 and 5.

##### Content validity of the Likert scale questionnaire

The 20 expert panelists (subject matter experts, SMEs) evaluated each statement on the 15-item Likert scale to determine if it is “essential or not necessary” for gauging participants' needs for health education. An item’s level of content validity increases with the panelists’ level of agreement that it is essential. For every Likert scale statement, the content validity ratio (CVR) was calculated. Values greater than 0.0 signify that a minimum of 50% of the SMEs concurred that the question is crucial and the closer the CVR is to + 1, the higher the content validity [20]. The content validity of the Likert questionnaire was found to be high for various statements, as indicated by the CVR range of 0.8–1.0. The content validity index (CVI) was computed to assess the test’s overall content validity. The average CVR scores across all Likert scale statements made up the CVI. The content validity increases with the CVI's proximity to + 1. The CVI of the Likert scale questionnaires was 0.9133. This indicates that the test's content validity was good and there was a high degree of agreement among the panelists [21].

##### Face validity of Likert scale questionnaire

Prior to the survey, the face validity of the questionnaire was evaluated. The question of whether a test seems to measure what it is intended to measure is known as face validity. When a questionnaire appears to be measuring what it is intended to, it is said to have good face validity [21]. To evaluate the questionnaire’s face validity, 30 individuals—nurses, doctors, and diabetics—were selected using the convenience sampling method and participated in a pilot study. They were asked to examine the items and determine whether or not they were appropriate for gauging the participants' needs for health education. They were asked to respond to the following inquiries: Are the questionnaire’s items pertinent to the subject being measured? Do individuals with diabetes seem to have a need for health education that can be measured using this particular method? Does the measure appear to be suitable for identifying the health education requirements of the participants? Reviewers of the questionnaire concurred that it was valid after a few small changes, such as adding some explanation phrases to the responses on the 5-point Likert scale.

### Data collection

Following an appropriate explanation of the study's objectives to participants, a group of trained nurses—including those who had participated in the face validity assessment of the questionnaire—gathered the required data between October 1, 2023, and November 16, 2023, using a personal interview questionnaire in Arabic language, for each participant (*n* = 303). The study participants—people living with diabetes—were advised to select one response from the 5-point Likert statements based on the importance of the statement as a health education need from their point of view.

To assess test–retest reliability, the test was given to the first 40 patients again via phone consultation on November 17, 2023.

### Statistical analysis

Data analysis was done using the Statistical Package for Social Sciences (SPSS Inc. Version 18.0. Chicago, USA). Descriptive statistics were conducted to determine the mean and standard deviation of continuous variables. Regarding continuous variables, a group *t*-test was applied. The 5-point Likert scale was converted into scores that ranged from 1 for strongly disagree to 5 for strongly agree for each Likert scale statement and from 15 to 75 for the entire Likert scale because every statement on the Likert scale was positive. The Likert scale’s internal consistency was evaluated using Cronbach’s alpha. Test–retest reliability was assessed using Pearson's correlation. Exploratory factor analysis (EFA) was conducted to assess the Likert scale’s construct validity.

## Results

### Baseline characteristics and Likert scores of the study group

The survey was carried out from October 1, 2023, until November 16, 2023. There were 303 participants in total. The survey had a 100% response rate. Of the total, 123 (40.6%) were men and 180 (59.4%) were females (Table 1). Their ages ranged from 18 to 94 years old, with a mean of 55.9 ± 12.9 years. The male age, 57.4 ± 12.8 years, did not differ significantly from the female age, 54.9 ± 12.9 years (*t*-value = 1.590 and *P* = 0.113).

**Table 1:** Sociodemographic characteristics of the diabetic participants, Jazan Armed Forces Hospital, 2023 (*n* = 303)

Sociodemographic characteristics	Number (percent)^a^
Gender	
Male	123 (40.6)
Female	180 (59.4)
Age in years	55.9±12.9^b^
Age category	
18–	7 (2.3)
25–	107 (35.3)
≥ 50 years	189 (62.4)
Duration of diabetes (years)	7.0 (3–13)^c^
Marital status	
Single, divorced, or widowed	61(20.1)
Married	242 (79.9)
Occupational status	
Not working or retired	212 (70.0)
Working (military or civilian)	91 (30.0)
Educational status	
Illiterate or read and write	86 (28.4)
Primary, preparatory, and secondary school	140 (46.2)
University degree	77 (25.4)
Type of diabetes	
Type 2	287 (94.7)
Gestational diabetes mellitus	7 (2.3)
Don’t know	9 (3.0)
Best source for getting information about diabetes	
Social media	15 (5.0)
Diabetes awareness events	1 ( .3)
Brochures and posters	5 (1.7)
Medical professionals	264 (87.1)
Social media and medical professionals	18 (5.9)

^a^Number and percent unless mentioned otherwise

^b^Mean and standard deviation in parenthesis

^c^Median and interquartile range (IQR) in parenthesis

Seven (2.3%) of the 303 patients were under 25, 107 (35.3%) were between the ages of 25 and 50, and 189 (62.4%) were over 50. Of the total 303 participants, 212 (70%) were unemployed or retired and 242 (79.9%) were married. Concerning education, 86 (28.4%) were illiterate or could read and write; 77 (25.4%) held a university degree; and 140 (46.2) were certified with a pre-university degree, such as intermediate school or higher. The duration of diabetes was not normally distributed, with a median of 7 and an interquartile range of 3–13 years. The study group consisted primarily of type 2 diabetics (*n* = 287; 94.7%), with pregnant females having gestational diabetes making up only 2.3%. Most participants (87.1%) believed that speaking with a medical professional is the best way to learn about diabetes. For 15 (5%) of the patients, social media was thought to be the best way to be informed about diabetes. Out of the 303 participants, only 6 (2%) believed that diabetes awareness events, brochures, and posters were suitable means of educating people about diabetes (Table 1).

The 5-point Likert scale was converted into scores that ranged from 1 for strongly disagree to 5 for strongly agree. The overall Likert scale scores fell between 15 and 75. The scores for the participant responses to various statements on the Likert scale were represented by the descriptive statistics shown in Table 2. The entire Likert scale scores had a mean of 61.99 ± 6.87 and ranged from 30 to 74.

**Table 2:** Likert scale scores among the diabetic participants^a^ (*n *= 303)

The statement (S), in my opinion, is important as diabetics’ health education need^c^	Range	Mean	SD^b^
S1: Risk factors and prevention of diabetes^.^	2.0–5.0	4.70	0.58
S2: Prediabetes (causes, symptoms, and treatment)	2.0–5.0	4.68	0.57
S3: General Information about diabetes (pancreas and insulin, causes of diabetes, diagnosis, symptoms, and types).	2.0–5.0	4.69	0.60
S4: HbA1c and normal blood glucose levels	2.0–5.0	4.75	0.50
S5: Treatment of hypoglycemia by the common oral agents	2.0–5.0	4.65	0.66
S6: Medical treatment by insulin (types, storage, sites of injection, and insulin pump)	1.0–5.0	3.33	1.30
S7: Diabetes and complementary therapies	2.0–5.0	2.95	1.13
S8: Acute complications of diabetes (hypoglycemia, diabetic ketoacidosis, etc.	1.0–5.0	4.59	0.72
S9: Chronic complications of diabetes, causes, symptoms, and treatment	2.0–5.0	4.69	0.55
S10: Importance of annual eye screening to prevent diabetic eye complications	2.0–5.0	4.68	0.59
S11: Diabetes and travel	1.0–5.0	4.66	0.68
S12: Diabetes and fasting	1.0–5.0	4.65	0.71
S13: Diabetes and pilgrimage	1.0–5.0	4.33	1.14
S14: Diabetes and work and driving	1.0–5.0	2.49	1.24
S15: Diabetes and school	1.0–5.0	2.15	0.93
Total Liker scale score	30.0–74	61.99	6.87

^a^Participants were encouraged to select one response for each 5-point Likert scale statement: strongly disagree, disagree, neutral, agree, and strongly agree

^b^ *SD* standard deviation

^c^Likert scale statements from S1 to S15

### Reliability of the questionnaire

The most common use of Cronbach’s alpha is the measurement of internal consistency, or “reliability”. The 15-item questionnaire got a Cronbach’s alpha of 0.832, indicating a high level of internal consistency. The final Cronbach’s alpha increased to 0.899 after the statements (*n* = 5) that would yield a larger Cronbach’s alpha if eliminated were removed. Statements 6, 7, 13, 14, and 15 were deleted. Table 3 shows the Cronbach’s alpha values for the final 10-item Likert scale questionnaire, which ranged from 0.883 to 0.899.

**Table 3:** The item-total statistics of Cronbach’s alpha for the final Likert scale after deleting redundant items

Likert scale items^a^	Scale mean if the item deleted	Scale variance if item deleted	Cronbach’s alpha if item deleted
S1: Risk factors and prevention of diabetes.	42.0528	16.507	0.884
S2: Prediabetes (causes, symptoms, and treatment)	42.0660	16.499	0.884
S3: General information about diabetes (pancreas and insulin, causes of diabetes, diagnosis, symptoms, and types)	42.0594	16.281	0.883
S4: HbA1c and normal blood glucose levels.	42.0000	17.219	0.889
S5: Treatment of hypoglycemia by a common oral agent	42.0957	16.425	0.890
S8: Acute complications of diabetes (hypoglycemia, diabetic ketoacidosis, etc.	42.1617	16.315	0.894
S9: Chronic complications of diabetes, causes, symptoms, and treatment	42.0594	16.857	0.887
S10: Importance of annual eye screening to prevent diabetic eye complications	42.0660	17.287	0.896
S11: Diabetes and travel	42.0891	16.783	0.897
S12: Diabetes and fasting	42.0924	16.740	0.899

^a^ *S* Likert scale statement (item)

After about a month from the initial assessment, the first 40 of the 303 participants retook the test (survey). The total Likert scores of those individuals (60.2 ± 4.9) were compared using Pearson’s correlation to see if there was any correlation with their initial measurement results (60.2 ± 4.7). The two measurements showed an extremely significant association (*r* = 0.864; *P* = 0.001. Thus, the survey's test–retest reliability is excellent, we might conclude.

### Validity of the questionnaire

#### Content and face validity

As previously stated in the “Section 2”, the questionnaire showed good face and content validity.

#### Construct validity

To evaluate the construct validity of the needs assessment questionnaire, exploratory factor analysis (EFA) was used. Utilizing the 10 Likert scale variables (items) that were obtained from the previously described Cronbach alpha analysis, factor analysis (also known as principal component analysis, or PCA) was performed. To determine whether the data were suitable for factor analysis, KMO, and Bartlett’s tests were performed first. The data are appropriate for factor analysis since the Kaiser–Meyer–Olkin sampling adequacy score was 0.870 (*P* < 0.001).

**Fig. 1:**
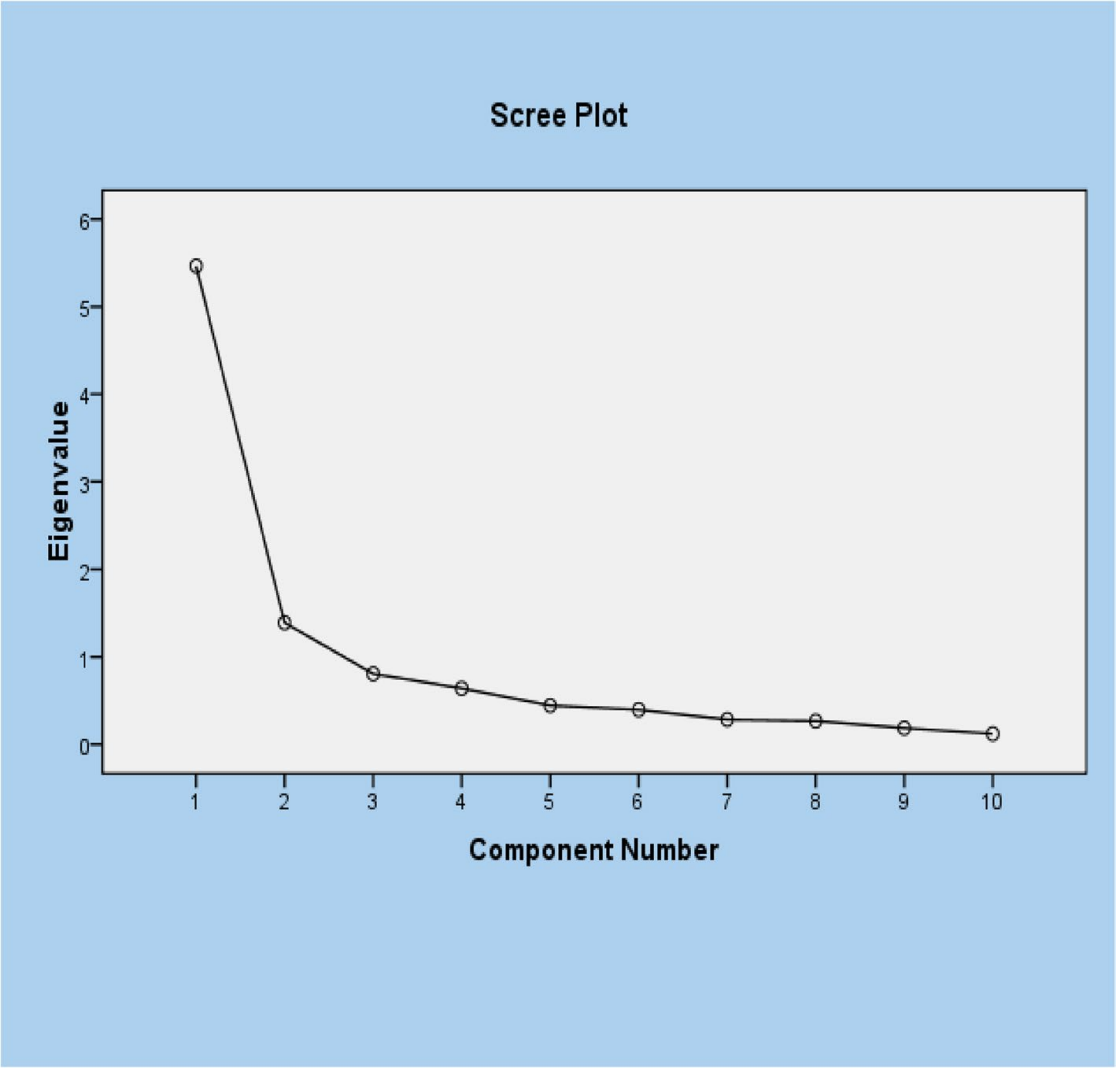
The scree plot of factor analysis: the relationship between component number and eigenvalues

Table 4 shows that the number of factors with eigenvalues greater than 1 is limited to two. In the factor analysis, both factors account for 68.6% (54.7 for Factor 1 and 13.9 for Factor 2) of the total loading variance. According to the factor analysis scree plot, Component 2 has an “elbow” joint. This is the tipping point where it might not be as advantageous to extract more components. We selected two components with eigenvalues greater than 1 (high-quality factors) using the scree plot, as shown in Fig. 1.

**Table 4:** Principal component analysis: total variance of extracted factors with an eigenvalue above 1^a^

Component	Initial eigenvalues			Extraction sum of squared loadings			Rotation sum of squared loadings		
	Total	% of variance	Cumulative %	Total	% of variance	Cumulative %	Total	% of variance	Cumulative %
1	5.46	54.666	54.666	5.46	54.666	54.666	4.17	41.721	41.721
2	1.39	13.906	68.572	1.39	13.906	68.572	2.68	26.851	68.572
3	0.80	8.071	76.643						
4	0.64	6.406	83.048						
5	0.44	4.438	87.486						
6	0.39	3.957	91.443						
7	0.28	2.833	94.276						
8	0.26	2.667	96.943						
9	0.18	1.848	98.792						
10	0.12	1.208	100.000						

^a^Extraction method principal component analysis

To determine the name of the component, the researchers examined the factor analysis component matrix, which displays Pearson’s correlation coefficients of different items (variables) with the two components that were extracted. Statement 3 (general information about diabetes) had the highest correlations with Component 1 while Statement 11 (diabetes and travel) had the strongest association with Factor 2. Thus, we may deduce that Component 1 is titled “General Information about Diabetes,” while Component 2 is titled “Diabetes and Travel”.

The findings of a rotated component matrix are shown in Table 5 to help identify the variables that have a strong correlation with each component. The variables are arranged in descending order based on how strongly they correlate with each component. Factor 1 (Component 1) exhibited noteworthy associations with Statement 1 (S1): Diabetes risk factors and prevention, S2: Prediabetes (causes, symptoms, and treatment), S3: General information about diabetes (disease causes, diagnosis, symptoms, types, pancreas and insulin), S5: Common oral medications for treating hypoglycemia; S4: HbA1c and normal blood glucose levels; and S8: Acute problems related to diabetes, such as hypoglycemia and diabetic ketoacidosis. On the other hand, S11: Diabetes and travel; S12: Diabetes and fasting; S9: Chronic complications of diabetes, causes, symptoms, and treatment; and S10: The significance of the yearly eye screening to prevent diabetic eye complications were the Likert scale items that were contributing more to Component 2 (Diabetes and travel). Table 5 shows the final standardized Likert scale items, which have excellent reliability and validity.

**Table 5:** Rotated component matrix: identification of the final Likert scale items that are highly correlated with the two extracted components^﻿a^

Likert scale items^a^	Component	
	1 (General information about diabetes)	2 (Diabetes and travel)
S1: Risk factors and prevention of diabetes	0.917	
S2: Prediabetes (causes, symptoms, and treatment)	0.884	
S3: General information about diabetes (pancreas and insulin, causes of diabetes, diagnosis, symptoms, and types)	0.853	
S5: Treatment of hypoglycemia by common oral agent	0.766	
S4: HbA1c and normal blood glucose levels	0.730	
S8: Acute complications of diabetes (hypoglycemia, diabetic ketoacidosis, etc.	0.549	
S11: Diabetes and travel		0.874
S12: Diabetes and fasting		0.860
S9: Chronic complications of diabetes, causes, symptoms, and treatment		0.657
S10: Importance of annual eye screening to prevent diabetic eye complications		0.549

^a^Extraction method: principal component analysis, rotation method: varimax with Kaiser normalization

*S* Likert scale statement (item)

## Discussion

Patient education has long been seen as a crucial element of effective diabetes care [13]. Diabetes education needs to be adapted to the demands of those living with the disease and take into consideration cultural sensitivity. There are many validated questionnaires in the literature for assessing patient knowledge and identifying knowledge gaps. However, no validated questionnaire exists for evaluating patient needs regarding health education from the perspectives of both patients and healthcare providers. Therefore, there is a pressing need to develop an assessment instrument based on scientific principles [12]. The current questionnaire was created utilizing a modified Delphi panel procedure.

Our 15-item scale has a good degree of internal consistency, as shown by the Cronbach’s alpha of 0.832. The internal consistency of the questionnaire increased to 0.900 when redundant items (*n* = 5) were removed from the Likert scale. This increase leads us to the conclusion that the questionnaire has a 90% internal consistency. The study’s 86% test–retest reliability further supported the high reliability of the instrument. Furthermore, the completed survey exhibits noteworthy construct, face, and content validity. Two factors (components) with eigenvalues greater than 1 were identified by factor analysis from the 10-item Likert scale: general information on diabetes and diabetes and travel.

General information about diabetes exhibited noteworthy associations with the following items in a descending ranking order based on the degree of the linear association with each item: diabetes risk factors and prevention, prediabetes (causes, symptoms, and treatment), general information about diabetes (disease causes, diagnosis, symptoms, types, pancreas and insulin), common oral agents for treating hypoglycemia; HbA1c and normal blood glucose levels; and acute problems related to diabetes, such as hypoglycemia and diabetic ketoacidosis. On the other hand, Diabetes and fasting; chronic complications of diabetes, causes, symptoms, and treatment; and The significance of the yearly eye screening to prevent diabetic eye complications were the Likert scale items that contributed more to Component 2 (diabetes and travel).

In the present study, the patients reported the highest educational needs for diabetes risk factors and prevention; prediabetes (causes, symptoms, and treatment); and general information about diabetes (disease causes, diagnosis, symptoms, types, pancreas and insulin). To prevent diabetes in the general population as well as in high-risk groups like people with impaired glucose tolerance and impaired fasting glucose, it is necessary to be aware of the risk factors. Diabetes can occur at a lower rate if people are aware of the risk factors that lead to the disease [22]. Research has revealed that persons who believe they are at risk for a disease are more prone to take action and adhere to recommendations made to lower their chance of getting the disease. Therefore, a great deal of work needs to go into educating individuals about diabetes risk factors that alter their susceptibility [23]. Also, general information about diabetes (disease causes, diagnosis, symptoms, types, pancreas, and insulin) was an important item in the first component, a result that is consistent with other studies [9, 24]. Patients and parents have been reported to have significant demands for health education regarding insulin, oral hypoglycemic drugs, and the most recent developments in diabetes care, such as continuous glucose monitors, islet cell transplantation, and artificial pancreas [24]. The demands for health education found in this study align with those found elsewhere. For instance, in order to determine the need for health education among adult individuals with diabetes, a qualitative study was carried out in Iran. The authors of the study reported on a number of related topics, including the need for disease recognition, identification of disease causes, and awareness of disease symptoms [12].

In a similar study, the educational needs of patients with type 1 diabetes and their parents were evaluated. The parents of the patients indicated that their top education needs were for complication management, continuous management, physical activity and exercise, and disease characteristics [24]. By increasing awareness, the majority of diabetes mellitus (DM) complications can be greatly avoided. The first step in managing a health problem, as well as in facilitating preventative and control efforts, is raising awareness of the condition and its treatment. Furthermore, knowledge of the nature and complications of the disease is necessary for adherence to therapy. On the other hand, increased rates of complications are a result of a lack of knowledge about diabetic complications [25].

Among the complications of diabetes, hypoglycemia is a frequent and potentially dangerous one [26]. It can cause arrhythmia, coma, impaired renal function, cognitive dysfunction, hemiplegia, and even death [27]. It is considered to be a significant barrier to blood sugar control, which can exacerbate or cause problems for patients and negatively affect diabetes care [28]. Hence for those who have diabetes, it is crucial that they receive this health-related knowledge. The common oral agents for the treatment of hypoglycemia include glucose tablets, glucose gel tube, 4 oz (1/2 cup) of juice, and 1 tablespoon of sugar, honey, or corn syrup) [29].

One of the participants’ demands for health education in the present study was about travel with diabetes, which made up the second component of our final Likert scale. Traveling can be viewed as an essential aspect of our lives, regardless of the purpose—business, pleasure, sports, or pilgrimage. Numerous travelers have diabetes as a result of the worldwide diabetes epidemic. Even for those who have diabetes under control before their trip, the most typical obstacles are changes in food, activity, time zones, surroundings, and stress. Family doctors are in the best position to offer preventive health care for travelers with diabetes, and counseling is crucial for potential travelers with diabetes to avoid complications [30, 31].

In this study, a need was expressed for education regarding fasting throughout Ramadan. Family physicians should be proactive in their approach when consulting patients who have diabetes before Ramadan [32]. Structured education and support for safe fasting are useful in managing diabetes during Ramadan [32, 33]. The study participants were also in need of health education about chronic complications of the disease, the 3rd item of the 2nd factor of the final Likert scale. It should be mentioned that persons with diabetes are thought to have an unmet demand for health education regarding the chronic complications of their disease [34]. Additionally, one of our participants’ educational demands was the significance of yearly eye exams, which ranked as the fourth item of Component 2. This is particularly significant given that diabetic retinopathy is a major worldwide public health concern, and screening for it can save lives by reducing vision impairment [35].

Consistent with previous research, the majority of participants in this survey believed that consulting a medical practitioner is the best way to learn about diabetes [36]. Social media was considered the most effective way by 15 patients (5%) in the current study, and 18 patients (5.9%) believed that both healthcare professionals and social media are the best sources of information about diabetes. Other research has also highlighted the role of social media as a useful tool for diabetic self-care management improvement and health education [37].

### Strengths and limitations

#### Strengths

(1) Appropriate sample size. (2) There is no selection bias because the study sample was chosen at random. (3) The study sample, 303, was greater than the minimum needed for factor analysis (the minimum number needed for principal component analysis should be at least 200). (4) The study group, which consisted of individuals with diabetes, was chosen from all outpatient clinics across several departments at JAFH. (5) Researchers, data collectors, and patients all participated in the assessment of the questionnaire’s face validity. For determining patients’ needs, we may infer that the questionnaire is standardized and useful in both practice and research.

#### Limitations

The study only covers one hospital in the Jazan region.

## Conclusion

In conclusion, the final 10-item Likert scale is quite valid and reliable for identifying the demands of participants who have diabetes. The standardized questionnaire can be used in research and practice to determine what health education needs people have. Furthermore, policymakers, decision-makers, and healthcare providers can use our findings about the need for health education as a guide when developing health education programs. These programs should focus on the two identified aspects of the Likert scale: basic diabetes information and traveling with diabetes.

## Data Availability

The datasets used and/or analyzed during the current study are available from the corresponding author upon reasonable request.

## Abbreviations

CVI: Content validity index
CVR: Content validity ratio
DM: Diabetes mellitus
DSMES: Diabetes self-management education and support
EFA: Exploratory factor analysis
HbA1c: Glycosylated hemoglobin
JAFH: Jazan Armed Forces Hospital
KMO: Kaiser-Meyer-Olkin test
PCA: Principal component analysis
SMEs: Subject matter experts
SPSS: Statistical Package for the Social Sciences
